# Altered Images: Understanding the Influence of Unrealistic Images and Beauty Aspirations

**DOI:** 10.1007/s10728-016-0327-1

**Published:** 2016-07-18

**Authors:** Fiona MacCallum, Heather Widdows

**Affiliations:** 1 Department of Psychology, University of Warwick, Coventry, CV4 7AL UK; 2 Department of Philosophy, University of Birmingham, Edgbaston, Birmingham, B15 2TT UK

**Keywords:** Beauty ideals, Digital modification, Body image, Media

## Abstract

In this paper we consider the impact of digitally altered images on individuals’ body satisfaction and beauty aspirations. Drawing on current psychological literature we consider interventions designed to increase knowledge about the ubiquity and unreality of digital images and, in the form of labelling, provide information to the consumer. Such interventions are intended to address the negative consequences of unrealistic beauty ideals. However, contrary to expectations, such initiatives may not be effective, especially in the long-term, and may even be counter-productive. We seek to understand this phenomenon of our continued aspiration for beauty ideals we know to be unreal and even impossible. We draw on our respective disciplines to offer psychological and philosophical accounts for why this might be. We conclude that beauty ideals are deeply embedded in our aspirations, practices, and in our constructions of ourselves. Given this, it is not surprising that simply increasing knowledge, or providing information, will be insufficient to challenge them.

## Introduction

In this paper we consider the impact of digitally altered images on individuals’ body satisfaction and beauty aspirations. The proliferation of altered images is such that it is now standard for print and on-line images to be altered in some way: from relatively minor retouching (whitening teeth and eyes, smoothing wrinkles, and erasing blemishes) to more dramatic modification (elongating limbs and slimming waists, thighs and arms). We draw on the psychological literature to outline current understandings of the relationship between images of ideal (particularly thin) bodies on body image and the ideal body to which we aspire. We then consider interventions which seek to counter the influence of such altered images—those of media literacy and labelling—and focus on the troubling finding that knowing that images have been altered does not make us less likely to aspire to the ideals they represent. On the contrary it seems that labelling images as “manipulated” or “enhanced” may even increase our desire to aspire to the ideal portrayed. We seek to offer possible explanations for why we continue to desire to attain such ideals, even when we know that they are not real. We draw on our respective disciplines to offer a psychological and philosophical account for the allure of such beauty ideals.

## Digital Images and Body Image

From a sociocultural perspective on the formation of body perceptions and satisfaction, viewing of media depictions is an influential route of transmission of cultural ideals of beauty [10]. Extensive research supports this, with exposure to visual mass media depicting idealised bodies being associated with body image disturbance in both experimental and correlational studies [9].^1^ Although most frequently investigated in adolescent girls and young women, similar relationships are also seen in the midlife stage; viewing of media featuring “ageing beauties” (who have the body shape and size of younger women) predicted disordered eating, greater discrepancy between actual and desired body size, and stricter food choices in women aged 30–65 [15]. This is not to say that images are passively and uncritically received [11]. Rather, images are interpreted, critiqued, rejected and renegotiated as individuals and groups. This said, an increasingly globalised and homogenised beauty ideal is emerging, which gives particular prominence to thinness and youth. The increasing visual emphasis on these attributes over the last few decades has been paralleled by rises in rates of body dissatisfaction with both women and men feeling unhappy with their physical appearance [24]. Negative body image can be viewed as a core aspect of psychological wellbeing, relating not just to appearance-changing behaviours such as dysfunctional eating behaviours, but also to general emotional difficulties such as distress and depression [20], making it an issue of real concern.

Digital alteration means that increasingly the images with which we are bombarded are ever more idealised and unreal, and this exacerbates the problem by setting ever higher expectations of what it is to be normal, good enough, or perfect. Airbrushing to remove imperfections, whiten teeth, elongate and narrow limbs, slim waists, increase breasts, is not only accepted but expected in the fashion and entertainment industries [6]. Low-cost technology means that modification is not confined to the photographer’s studio but can be implemented via apps in order to “improve” photos posted on social media, exhorting users to “Get Instagram ready!” The ubiquity of these techniques can be regarded as a factor in creating increasingly unrealistic beauty aspirations, leading to negative consequences of increased body dissatisfaction with its adverse implications.

## Revealing the Unreal

In response a number of interventions have been suggested, and we consider two of these: (a) education programmes in schools including components focusing on media literacy and the artificial nature of images (see e.g. [12]), and (b) the explicit labelling of images as digitally altered.

Several types of school-based interventions aimed at improving body image have been trialled, and a meta-analysis found that those which were effective included content relating to media literacy and the way in which media images can be manipulated, supporting the first proposed approach [29]. However, the effect sizes of these programmes were only small, and improvements were not always maintained post-intervention [1]. One difficulty may lie in our ability to know when a photograph has actually been altered. Although computational methods can be designed to detect image tampering, it is not so easy with the naked eye [7]. A recent experiment using real-life photographs showed that when presented with an airbrushed image (teeth whitened, spots/wrinkles removed, and so on) and asked to decide if it was original or digitally altered, more than half of the participants did not detect it was manipulated, and of those who did, many were unable to identify which aspect had been changed [19]. Furthermore, people’s belief about the extent of image manipulation in general did not improve their ability to detect and locate the alterations. Importantly then, simply being aware that a lot of images are modified does not mean we are any better at spotting them in the real world, suggesting that media literacy training may not be enough.

If knowing that images generally are manipulated is not sufficient, perhaps being told which specific images have been altered would be beneficial. This would have the additional advantage of reaching all individuals interacting with the media rather than just those still at school. In both Israel and France, it is now a legal requirement for advertisers to disclose when photographs of models have been digitally modified. Australia has a Voluntary Code of Conduct for the fashion, media, and advertising industries, requesting that disclaimer labels be included on altered images, and similar policies have been put forward in other countries such as Norway and the UK. But do such strategies work in reducing the idealisation of the images, and thus the negative effects on our self-perceptions?

Some initial research on the effects of labelling was promising with an Australian study finding that including a label warning that an image had been digitally altered reduced the level of body dissatisfaction created by viewing a thin ideal image [22]. However, a growing number of studies have found no amelioration of the negative effects of media images by labelling, and in fact the opposite may be the case. An experiment by Bissell presented one group of women with information about the general use of digital enhancement in the media and then with images of models in swimsuits with the tagline “the image below has been digitally manipulated to enhance the model’s appearance” [2]. The results showed that, compared to a second group of women who viewed the same images with no statement and no prior information, those in the experimental group reported a *greater* desire to look like the models, and rated the models as more attractive. This “boomerang effect”, where awareness of digital modification increases rather than diminishes the influence of the images has also been found using retouched images of normal people, to control for the effect of the thin-ideal model [14]. In this study, male and female adolescents viewed photographs of same gender young adults that were either not retouched, were retouched to remove blemishes and subtly improve body contours, and labelled as so, or were retouched and not labelled. Physical self-esteem decreased and objectified body consciousness increased following exposure to the images only in those adolescents who were informed about the digital alteration.

These undesired effects may also be durable across time. A single exposure to a thin-ideal image with a disclaimer label (worded in the same way as the then-proposed French law—“this image has been altered to modify a person’s bodily appearance”) increased accessibility to negative thoughts, which was used as an implicit measure of an adverse cognitive-emotional response, immediately after viewing [21]. Importantly, these effects were also seen when participants viewed the image again, with no disclaimer this time, both 2 weeks and 2 months later. The implication from the research is that if an image is considered desirable, digital alteration disclaimers are not helpful and may actually be harmful. One study found that a specific information label attached to images of thin media models. i.e. “these models are underweight”, did have the desired effect of reducing negative body perceptions [27]. As a policy strategy, the feasibility of asking the advertising and fashion industry to adopt labels worded in this way might be questionable as it would be likely to have negative commercial consequences, although it could also have the positive effect of discouraging the use of underweight models.^2^

Such studies have limitations in what they reveal, and the impact of images is cumulative, and dependant on social and cultural context. But, and this is the point, the ubiquity of increasingly unrealistic digital images does feed into our beauty ideals and aspirations, and it seems that we continue to hold digitally modified images as ideals even when we are told that they are not “real”. If this is the case then simply providing more information, or knowledge, is not sufficient. In the rest of the paper we suggest three possible explanations for what at first glance seems counter-intuitive, that knowing something is unreal—even impossible—does not stop us aspiring to it.

## Ideals, Comparisons and Ourselves

Before considering the question from a theoretical perspective, it is helpful to understand what is happening in terms of perceptual processing, i.e. what our senses do with the labelled images. Here, we draw first on Selimbegovic and Chatard who, suggested that disclaimer labels actually draw more visual attention to the image, leading to “deeper” processing of it [21]. The result is more focus on the semantics of the image, its meaning and implications, i.e. viewers are reminded that “being beautiful means looking like this”. This would also explain the durability of the effect whereby deeper processing involves more elaborate encoding in memory, and thus easier recall. A similar account by Tiggemann and colleagues was proposed for the findings of another study where specifically worded labels, such as “Warning: this image has been digitally altered to smooth skin tone and slim arms and legs”, were found to increase body dissatisfaction for women who had pre-existing high levels of the tendency to compare their appearance to others [25]. The authors suggested that the warning labels directed greater attention to the model’s body, and particularly those areas named as altered, than would be the case with a generic warning label or with no label at all. They went on to investigate this speculation using eye tracking technology, by presenting women with fashion advertisements depicting the thin ideal, and labelled with a specific disclaimer referring to target areas of the body, e.g. waist, or with a generic disclaimer stating that the image had been digitally altered, or with no disclaimer [4]. Measurements of the number and the duration of gazes towards the target areas of the models’ bodies found that women looked at these areas more often and for longer in both the disclaimer label conditions, and even more so in the specific than the generic label condition. This additional attention predicted an increase in body dissatisfaction for those who saw the specific warning labels. Thus, far from encouraging us to discount altered images, labelling seems to make us take more notice of them.

The finding that women high in social comparison were more likely to experience increased dissatisfaction when viewing specific warning labels ties in with one potential theoretical explanation [25]. Social comparison theory, as first put forward by Festinger, postulates that humans have a desire to self-evaluate, and that where no objective measurement of our attributes is possible, as with appearance, we do so through comparison with others [8]. Although to some extent, this seeking of information is a rational tactic when direct assessments are unavailable, the consequence is that our self-worth judgements depend on the chosen comparison target. “Upwards” comparisons, where we perceive the comparator as higher in the particular attribute will lead to more unfavourable self-evaluation. Social comparison has consistently been demonstrated as one of the processes determining body dissatisfaction [18]. When idealised media images are used as comparison standards, upwards comparisons lead to more negative perceptions of our own appearance, accounting for some of the influence of the media on body image [11]. The rationale for recommending disclaimer labels initially was that social comparison targets are chosen based on their self-relevance and their salience [23, 25].^3^ Therefore, awareness of digital enhancement should lead to less comparison and less consequent body dissatisfaction since the image is known to be artificial and so not a relevant and salient target [4]. However, paying more attention to the image as discussed above seems to have the opposite effect of increasing the felt relevance, and encouraging upwards comparisons. Effectively, the enhanced attention reinforces our conception of the ideal, and we are reminded that we do not match up. Thus, labelling images as digitally altered exacerbates negative social comparisons with ideal images, which in turn exacerbates criticism of our own appearance.

A second framework which could explain why digital modification affects us negatively even when we are aware of it is that of self-discrepancy theory [17]. This proposes that individuals hold self-perceptions in three domains: the “actual” self (the attributes we believe we have); the “ideal” self (the attributes we aspire to have); and the “ought” self (the attributes we believe we *should* have). When discrepancies arise between these perceptions, it can lead to negative emotions and cognitions. Specifically, discrepancy between the actual self and the ideal self can result in dejection-related emotions such as disappointment and sadness, whereas discrepancy between the actual self and the ought self can result in agitation-related emotions such as anxiety and guilt. These types of discomfort lead people to engage in behaviours designed to resolve the discrepancies. Research on media influences on body satisfaction has focused mainly on the actual-ideal discrepancy and how media imagery can affect the formulation of the ideal self, making it more unattainable thus increasing distress, and making appearance-changing behaviours such as eating disorders more likely [11]. However, viewing idealised media can also increase the discrepancies between the actual and the ought self, resulting in behaviours such as restricted eating in the presence of others, in order to give the impression to other social agents that one is trying to achieve the appearance we believe society obliges us to have [15]. The labelling of images as digitally altered could affect the ideal self, with the processes of increased attention and deeper processing leading to further internalisation of the attributes of the image as ones we would like to possess. Equally though, the manipulation could be interpreted as being considered the socially desirable image, and therefore possessing attributes we ought to have. Even when we know the model does not look like that, the pressure on the ought self could put an obligation on us to try to emulate the image, and make us feel guilty when we do not.

Taken together then, there are psychological accounts which offer some reasons for why knowing that images are unreal may do little to stop us seeking to attain them, and in fact may further reinforce and embed unrealistic beauty ideals.

## Beauty as an Ethical Ideal

Turning from the psychological literature to the philosophical, we can explain the continued power of beauty ideals, however unreal, if we recognise that, at least for some women in some instances, beauty ideals are functioning as ethical ideals. By this, we mean that the extent to which a women conforms to the beauty ideal determines how morally good she judges herself (and others) and how she evaluates actions as right and good, opposed to wrong and bad.^4^ The use of moral language here we regard as accurate and significant and not simply a matter of language. “I *should* go to the gym” because I *should* improve, is moral in that it is about what is required to be judged good (or good enough).^5^ If this is the case then it is not surprising that knowing that beauty ideals are unreal and unattainable does nothing to reduce the wish to attain such ideals. That there are moral and ethical elements in beauty ideals, standards and discourses is overtly the case. Many people judge themselves according to their success and failure in beauty terms. This is true both with regard to achieving long-term goals—we judge ourselves successful when we’ve attained some aspect of the ideal (reached our goal weight, filled our wrinkles or firmed our thighs)—and in terms of daily habits and practices—we are successful when we make it to the gym, stick to the diet, get a manicure or undergo some procedure. Success here is not just aesthetic success (although it may be that too), but moral. For instance, when we deem ourselves successful for engaging in many of these practices the success is only moral: sticking to our calorie count for the day or making it to our exercise class has very little impact upon how we look in the short term and the data on diets suggests that very few of us dramatically change our size over the long term. However, meeting the goal is less important than engaging in the practices, even though we may never reach the desired goal (and we know that it is unlikely), on a day-to-day basis we can still succeed.^6^

That beauty is functioning as an ethical ideal—providing values and standards against which we judge ourselves and others—is particularly clear when we consider what it means to “fail”. Beauty failure results in explicit moral judgements of culpability and responsibility, making beauty failures effectively equivalent to vices. That this is the case is hinted at in the language of beauty as employed by both women and the beauty business (advertisers and women’s magazines): be “your best self”, “the best you can be”, “it’s still you, but the best version of you”, “the real you”. Language such as this directly reveals and communicates the ethical nature of the beauty ideal. You *should* strive for that best, real, you; you *ought* to invest in yourself, because “you’re worth it”, “you deserve it” and “you owe it to yourself”. The converse is also true. To fail to engage is to admit or accept that “you’re not worth it”: “You let yourself go!” Unpacking the implications of this reveals the moral assumptions underpinning the framework: It implies not only that you *should* not have let yourself go, but more than this, that it was a bad thing to do and you *should* work to address your failure. Appearance then becomes a proxy for, and intimation of, character and value: thinness and grooming shows competence and efficiency, and scruffiness and dishevelment reveal inner turmoil or distress, and not dressing appropriately is a failure of respect (for the self or others). Such judgements are routinely and constantly made, read directly from appearance, and are moral. Effectively they are character assessments of virtues and vices. And just as we regard success as virtuous, shame and disgust attach to failure.^7^

Further, like other ethical ideals, the beauty ideal purports to deliver the goods of the good life: material goods, relational goods and lifestyle goods. Essentially the message is that if you conform to the beauty ideal, become better (thinner, firmer, smoother, younger) you will be rewarded with these goods: “That we’re desperate to be seen as fit and energetic and young and attractive makes sense when we are told on so many tacit and overt levels that we will find neither work nor sexual partners without these attributes; moreover, we are fated to lose both if we don’t retain at least the superficial vestiges of the original assets”.[3, p 49] While the evidence is somewhat contested there is significant empirical evidence which suggests that beauty does deliver at least some of the goods of the good life. The beautiful, for the most part, do better than those who are not beautiful; and although the differential is not dramatic nor is it negligible [13].

To illustrate consider a few statistics. A recent large UK study suggested that tall men and slim women are relatively significantly better paid than those who are not.^8^ This was reported in the popular media, somewhat dramatically, as: men earn £1600 a year for every 2.5 inches; and women genetically predicted to be two stone heavier are “losing out” by £3000 a year [5]. Even if we regard the causal genetic claims with some suspicion (especially when it comes to weight which is strongly linked to socio-economic factors and to possible discrimination and bias, something which the researchers themselves note), being tall as a man and being thin as a woman is likely to lead to some material advantage. Hamermesh draws the evidence from many empirical studies on this together and concludes that there is a “3 or 4 % premium for good looking workers” [13, p 47] and a greater difference between unattractive and attractive.^9^

However, even if approximating the beauty ideal does deliver some of the goods of the good life, striving for the ideal is important, even if it is not attained (and does not deliver any material goods), and even if it is unattainable. Recognising that the beauty ideal drives and influences behaviour—irrespective of the goods delivered—helps us understand why it is such a powerful ethical ideal. The beauty ideal promises the possibility of perfection, or at least improvement: The perfect self remains beyond and in the future, an ideal to approximate, a possibility to be strived for and aspired to, and to be worked at. That it is unattainable does little to reduce the power of the ideal or our commitment to it. Being perfectly good, or humble, are equally unattainable, but yet ideals which have been striven for in many contexts. Understanding the power of ideals goes some way to explaining why, even when we know images are re-touched, we continue to judge ourselves against them and wish to attain the ideal they promise. The perfect image on the page feeds into our imaginings of the perfect ideal we are seeking to embody. Like other ethical ideals, the beauty ideal not only holds out a promise of perfection (whether understood as being perfect, or simply becoming better, normal or improved), but also offers daily habits and practices to help us attain it and by which to structure our lives. Thus, not only does it provide a value framework, but dictates tasks, skills and knowledge: To be good you must engage in daily practices (do a good turn every day); to be beautiful you must also engage in daily practices (those of routine maintenance, from hair removal to exercise). As such, like other ethical ideals it provides both long-term goals and mundane techniques which together structure and give meaning to day-to-day existence.

By both promising goods and sanctioning failure, the beauty ideal engenders commitment from those who fall under it. It is this emotional commitment and investment in the ideal (manifested in the extent to which we judge ourselves and others by it) that helps to explain why images which present us with instances of the perfect ideal do not lose their power simply because we know they are digitally re-touched. Our imaginings of our perfect—or improved or better or good enough—self, the end point of the beauty ideal for which we are striving, has very little to do with what is actually achievable or likely to be achieved. Indeed, as we age the possibility of attaining the beauty ideal becomes ever less likely, but this does not mean that we reject the ideal or stop engaging in beauty practices. As technological interventions become more normalised and more accessible, so it becomes possible to attain some aspects of the beauty ideal, into middle and old age and increasingly pressures to conform to the beauty ideal, which once stopped or lessened at marriage or the menopause, now continue. Consequently as we age and fall further from the ideal we may feel more pressure to engage, rather than less.^10^

## Conclusion

In sum then, there are both psychological and philosophical accounts which offer some explanation for the initially surprising conclusion that drawing attention to digitally altered images may not, as one might expect and hope, reduce the aspiration to attain contemporary beauty ideals (to be thin, shapely and youthful). We are not claiming that from this we should conclude that there are no forms of intervention with regard to media literacy which might be effective in altering beauty ideals, for instance, some suggest that a greater diversity of models of beauty might widen the beauty ideals to which we aspire. However, we are suggesting that beauty ideals cannot be easily challenged by such interventions. Beauty ideals are culturally constructed and are carriers of meaning and value; accordingly if they are to be challenged, the extent of their ethical nature, and the way in which individuals actually make use of images for their own imaginings, needs to be recognised.

## Footnotes

1 That is not to say that this is a simple causal link. The extent to which an individual’s self-image is affected by exposure to visual media will also depend on personal characteristics, e.g. those who already have high levels of internalisation of sociocultural standards of appearance may be more vulnerable to media influence.

2 The use of excessively thin models in itself raises ethical concerns, and is regulated against in some countries including France, Israel, Italy and Spain.

3 In Festinger’s original conceptualisation, social comparison was premised on peer comparison; however, more recent adaptations suggest that the strength of the cultural ideals of attractiveness mean that people will not dismiss models and other idealised figures as comparison targets. Because women know they will be judged against these standards, the idealised images become relevant targets.

4 This argument is developed by one of the authors as part of a larger project [28]. The claim that beauty ideals are functioning as ethical ideals is in part a virtue based one, that for many to conform to the beauty ideal is considered virtuous (this is true externally, when character-traits are judged from appearance, and internally when we consider ourselves “good” when we diet or go to the gym or all manner of beauty practices). Many judge actions morally “good” or “right” when they are likely to deliver a better or more ideal body.

5 “Should” here is a moral “should”. It is not the prudential should of “you should write a will”, or the preference should of “you should have this pizza, its great!”.

6 For instance, in her analysis of Weight Watchers, Cressida Heyes argues that part of the logic of Weight Watchers is the recognition that we have to keep going and keep striving. It is never done, and when we stop dieting, “we mourn not only the loss of the future thin self that, even if not attained, can always be looked forward to, but also the loss of a forum in which, however conditionally, we might be helped to take care of ourselves”. [16, p 87]. Thus the ethical ideal is manifest in habits and practices as well as in aspirations and goals.

7 Narratives of “shame” appear across beauty practices; fat-shaming being the most obvious example, but also when it comes to botched surgery, visible body hair, and aging. This account fits well with the “ought self” and suggests that there are parallels between this psychological understanding and accounts of the self under the beauty ideal as an ethical ideal.

8 Using UK Biobank data of 119,669 participants, researchers aimed to study the causal effects of difference on stature and BMI against measures of economic status. It found that short stature and higher BMI were “observationally associated with several measures of lower socioeconomic status” [26].

9 Hamermesh presents a case from many studies that there is a beauty premium and an ugly penalty. For men this is 17 % difference in earnings between attractive men and unattractive men, and 12 % between attractive women and unattractive women. [13, p 46].

10 Contrary to some previous assumptions it may be that pressure to conform to beauty ideals is not a preserve of the young but extends into middle and old age.
